# REACTOR: REgulon Activity analysis and Comparison Tool for single-cell transcriptOmics Research

**DOI:** 10.1093/bioinformatics/btag203

**Published:** 2026-05-04

**Authors:** Markus Lindén, Sebastian I Zúñiga Norman, Tommi Välikangas, Sini Junttila, Tomi Suomi, Kalle T Rytkönen, Laura L Elo

**Affiliations:** Turku Bioscience Centre, University of Turku and Åbo Akademi University, FI-20520 Turku, Finland; Turku Bioscience Centre, University of Turku and Åbo Akademi University, FI-20520 Turku, Finland; Turku Bioscience Centre, University of Turku and Åbo Akademi University, FI-20520 Turku, Finland; Turku Bioscience Centre, University of Turku and Åbo Akademi University, FI-20520 Turku, Finland; Faculty of Science, University of Turku, FI-20520 Turku, Finland; Turku Bioscience Centre, University of Turku and Åbo Akademi University, FI-20520 Turku, Finland; Turku Bioscience Centre, University of Turku and Åbo Akademi University, FI-20520 Turku, Finland; Institute of Biomedicine, University of Turku, FI-20520 Turku, Finland; Turku Bioscience Centre, University of Turku and Åbo Akademi University, FI-20520 Turku, Finland; Institute of Biomedicine, University of Turku, FI-20520 Turku, Finland

## Abstract

**Summary:**

We introduce REACTOR, a computational tool designed to detect differential activity of transcriptional regulators and their target genes (regulons) in single-cell RNA-sequencing data. It expands the currently available framework for regulon analysis by introducing a robust statistical test to detect differential regulon activity between conditions, such as disease versus control, with multiple replicates. By contrasting different conditions, REACTOR enables identification of key condition- and cell type-specific regulons. To demonstrate the use of REACTOR, we illustrate its performance in a publicly available COVID-19 dataset.

**Availability:**

REACTOR R-package together with an implementation vignette are available at https://www.github.com/elolab/REACTOR.

## 1 Introduction

Single-cell RNA-sequencing (scRNA-seq) has revolutionised transcriptomics by enabling comprehensive and unbiased interrogation of cellular processes at the level of individual cells. Central in the study of cellular functions is understanding cellular identities, their development, and gene regulatory networks (GRNs) explaining their transcriptional states and state transitions (Badia-I-Mompel *et al*. 2023).

The most notable and widely used tool for studying the GRNs utilising scRNA-seq data has been the Single-Cell rEgulatory Network Inference and Clustering (SCENIC) framework (Aibar *et al*. 2017), which allows GRN inference and motif discovery analyses, as well as predictions of key cell type-specific regulators and their direct target genes, known as regulons. There are also methods that enable detection of differential GRN activity from scRNA-seq, such as DiNiro (Oubounyt *et al*. 2023) and Sccomp-Reg (Duren *et al*. 2021). However, similar to SCENIC, these tools do not support testing the differential GRN or regulon activity across replicates. Thus, there is a demand for a tool that can efficiently detect statistically significant differences in regulon activity between conditions, such as disease versus healthy states, or across other experimental groups.

To address this demand, we here introduce REgulon Activity analysis and Comparison Tool for single-cell transcriptOmics Research (REACTOR). REACTOR integrates SCENIC-derived regulon activities (Aibar *et al*. 2017) with reproducibility optimized statistical testing (ROTS) (Suomi *et al*. 2017) to detect differential regulon activity in scRNA-seq data with multiple replicates. The choice of method for differential analysis of omic data is critical, as traditional statistical approaches are often not optimal when applied to high-dimensional omic datasets (Mukherjee and Roberts 2005). ROTS has been shown to work well across various types of omic studies, including bulk and single-cell transcriptomics (Jaakkola *et al*. 2017, Soneson and Robinson 2018), proteomics (Suomi *et al*. 2017, Fröhlich *et al*. 2022), DNA methylation (Seyednasrollah *et al*. 2016), as well as ChIP-seq and ATAC-seq data (Faux *et al*. 2021). The integration of ROTS allows REACTOR to robustly reveal regulons that show statistically significant activity changes in a cell type-specific manner, providing insights into GRNs that are associated with specific cellular states.

To illustrate the use of REACTOR, we applied it to a publicly available scRNA-seq dataset of peripheral immune responses in patients with severe COVID-19 and healthy controls (Wilk *et al*. 2020). Using this case example, we demonstrate the core steps of REACTOR from identifying the most significant regulons to the basic visualization of the results. Additionally, to further validate the relevance of these results, we utilize a COVID-19 resource that gathers multiple gene expression studies on patients with SARS-CoV-2 (Välikangas *et al*. 2022) and existing literature. Overall, our study presents a new tool that holds potential for advancing our understanding of GRNs, especially in the context of case-control studies.

## 2 Materials and methods

**Figure 1:**
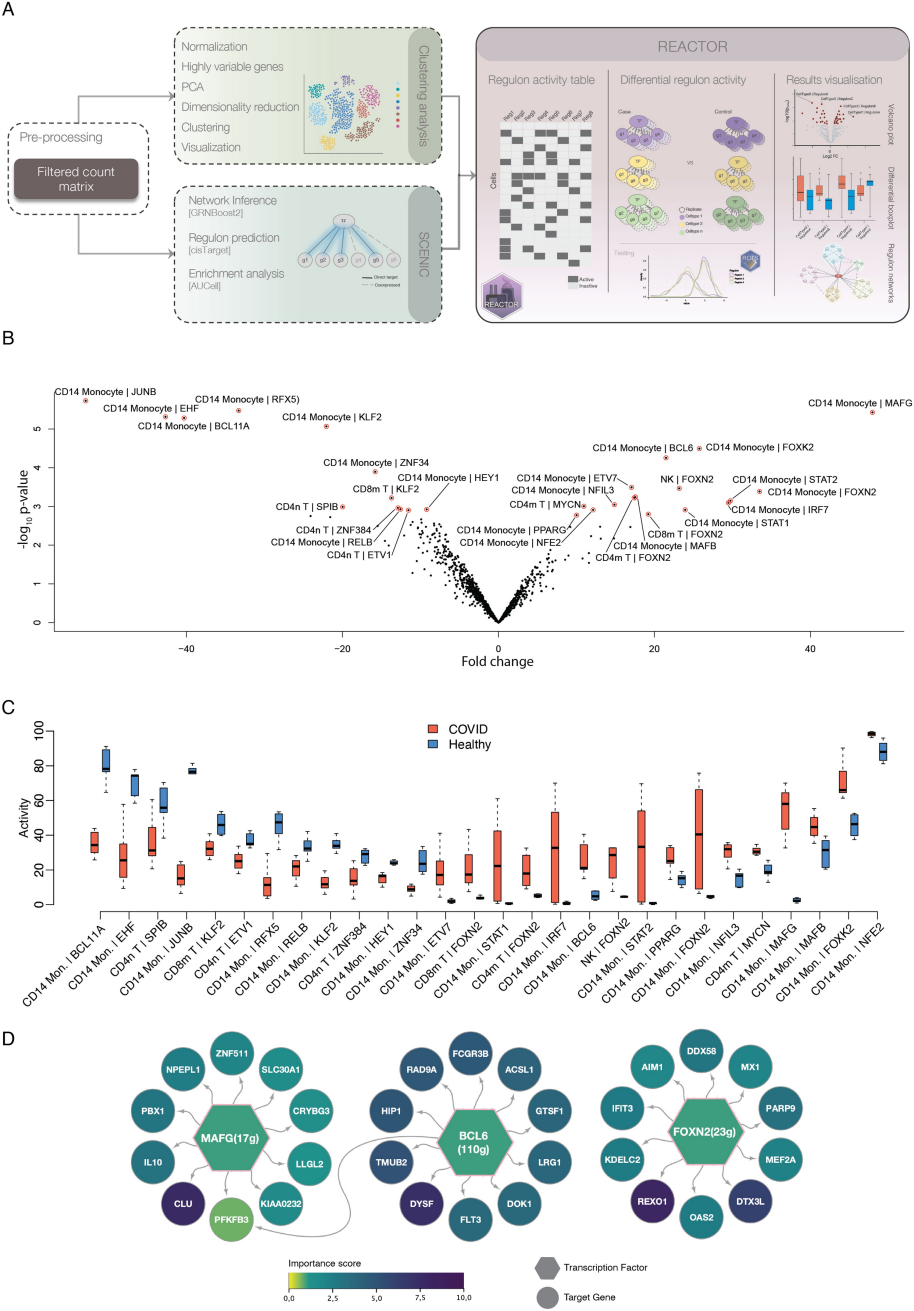
Overview of the REACTOR workflow with example results from a COVID-19 case study. (A) Schematic representation of the REACTOR workflow in relation to single-cell clustering and regulatory network inference as inputs. Workflow steps indicated by dashed lines are not the focus of this work, but specific outputs from both are required for REACTOR. SCENIC steps are based on (Aibar *et al*. 2017). The REACTOR workflow begins with a regulon activity table generated from binarized SCENIC output, contrasts varying numbers of replicates between conditions across cell types, and tests for differential activity using ROTS. Representative output examples are shown. (B) REACTOR detected regulons with differential activity using COVID-19 data as an example. The volcano plot depicts the fold change of the regulons on the x-axis and the -log10 p value on the y-axis. The 28 statistically significant regulons (FDR < 0.01) are highlighted with red circles and labeled. Regulon labels indicate the cell type followed by the regulon name. (C) Box plots showing the activities of the 28 regulons, with COVID-19 samples indicated in red and the healthy controls in blue. For each sample group, the regulon activity (y-axis) represents the fraction of cells in a cluster showing regulon activation. (D) Integrated network representation of the MAFG, BCL6, and FOXN2 regulons, identified among the most significantly upregulated regulons in COVID-19 and having the highest absolute regulon activity. As an example, only the top 10 target genes are displayed, based on the importance score from the initial SCENIC step reflecting transcription factor-target inference.

The overall REACTOR workflow is illustrated in Fig. 1A. The workflow begins with preprocessing and clustering of the scRNA-seq data (as described in Section 2.1), followed by GRN inference and estimation of regulon activities using SCENIC (Aibar *et al*. 2017; Section 2.2). REACTOR then constructs regulon activity tables for each cluster-regulon pairing across samples, which are subjected for the differential activity testing between conditions using ROTS (Suomi *et al*. 2017; Section 2.3). Finally, the results can be explored through visualizations and various prioritization criteria.

### 2.1 Clustering analysis

REACTOR aims to identify regulons that show statistically significant activity changes in a cell type-specific manner. Therefore, REACTOR requires a cell clustering as part of its input. As clustering is a standard step in scRNA-seq analysis workflows, it can be performed with the aid of different suites, e.g. Seurat (Hao *et al*. 2024), Coralysis (Sousa *et al*. 2025), or Scanpy (Wolf *et al*. 2018). These tools typically include steps such as normalization, highly variable gene detection, dimensionality reduction (e.g. PCA), clustering, and visualization (e.g. tSNE, UMAP) (Fig. 1A).

### 2.2 Gene regulatory network with SCENIC

The current implementation of REACTOR assumes SCENIC-derived regulon activities as input for differential regulon activity analysis. SCENIC itself is not part of the REACTOR tool but it is to be run separately prior to REACTOR. The SCENIC framework (Aibar *et al*. 2017) to infer regulon activities involves three main steps (Fig. 1A): (1) GRN inference using GRNBoost2 which calculates importance scores between transcription factors and their target genes. The importance score indicates how many times the regulator acts as a split factor in the tree structure of GRNBoost2’s underlying gradient boosting regression tree algorithm, inferred from the input data. This measures how well the expression level of the regulator can be used to predict the expression of the target genes. (2) Regulon candidate generation and posterior refinement using the RCisTarget method, and (3) quantification of regulon activity at cellular resolution using the AUCell method. Although not part of the three main steps, the optional SCENIC binarization of the regulon activity is required and used as an input for REACTOR. To make the SCENIC results directly comparable in REACTOR, a single SCENIC run for the whole study is used.

### 2.3 Implementation of REACTOR for differential regulon activity analysis

REACTOR performs statistical analysis over regulon activity profiles inferred by SCENIC in case-control studies and other experimental comparisons, including multi-group analysis (Fig. 1A). By assessing differential regulon activity in a cluster (cell type) -specific manner across replicates, REACTOR can compare regulon activity between experimental groups.

REACTOR is implemented as an R package and its usage requires three inputs: (i) a table containing the cell-level clustering information, where the first column specifies the cell ID and the second column the cluster name, (ii) a binarized regulon activity matrix generated by SCENIC [cells x regulons], and (iii) a study design table indicating the condition and replicate from which each cell originated, where the first column specifies the cell ID, the second column the replicate (e.g. donor 1, donor 2), and the third column the condition (e.g. treatment, control). See also https://github.com/elolab/reactoR for examples of input data structures.

First, REACTOR summarizes the three inputs with processData() function. This function creates a regulon activity table by combining the clustering information, binarized regulon activity matrix, and study design into a data frame, where rows correspond to cluster-regulon pairings and columns to samples. This regulon activity table summarizes the single-cell data to show, for each cluster-regulon pairing, the fraction of cells in the cluster showing regulon activation in a specific sample. To reduce noise from the data, minCells parameter (default 10) filters the regulon activity table, and represents the minimum number of cells (active or not) required for the subsequent differential analysis.

Second, REACTOR’s differentialActivityAnalysis() function performs differential activity analysis using ROTS (Suomi *et al*. 2017), with modified t- and F-statistics for two-group and multi-group comparisons, respectively. The parameter maxZeros is used for filtering data rows that contain more than the desired number of zeros before the differential activity analysis, with no default value. The resulting list of differentially active regulons can then be prioritized utilising false discovery rate (FDR), fold-change, or absolute activity score to select the most interesting regulons for further analysis.

### 2.4 COVID-19 case study

We utilised publicly available scRNA-seq data from a study of peripheral immune responses in seven patients with severe COVID-19, and six healthy controls (Wilk *et al*. 2020). The dataset contains 44 721 cells, with an average of 3194 cells per sample. We used the annotated clusters provided by the original study (Wilk *et al*. 2020), and the three steps of SCENIC were run with default parameters. For the REACTOR analysis, we set the minCells-parameter of the processData function to 10 and the maxZeros parameter of the differentialActivityAnalysis function to 3.

## 3 Results

### 3.1 Differential regulon activity in COVID-19

To demonstrate the use of REACTOR, we applied it to a publicly available COVID-19 scRNA-seq dataset (Wilk *et al*. 2020). In total, REACTOR detected 28 differentially active regulons at FDR < 0.01 across all cell clusters (cell types) (Fig. 1B). Visual inspection of the regulon activity with box plots supported their differential activity (Fig. 1C).

Several of the most significant regulons showed higher activity in COVID-19 cases compared to the healthy controls, including CD14 monocyte detected regulons MAFG, BCL6, and FOXN2 (Fig. 1B and C). MAFG (Utrero-Rico *et al*. 2021) and BCL6 (Utrero-Rico *et al*. 2021, Unterberger *et al*. 2025) are known master regulators of monocyte-specific responses in COVID-19, supporting the relevance of these REACTOR detected regulons.

The majority of the top regulons with significant differential activity were detected in CD14 monocytes, followed by a smaller number of regulons in other cell types such as natural killer cells (NK), CD4 and CD8 T cells, and B cells. Additionally, for one regulon, FOXN2, significantly higher activity was detected in all these five cell types. Increased COVID-19 associated FOXN2-gene expression has also been previously reported (Babu and Nobel 2022).

Finally, we used the COVID-19 resource (Välikangas *et al*. 2022) to investigate the expression of MAFG, BCL6, and FOXN2 in independent datasets (Figs. S1–S3, available as supplementary data at Bioinformatics online). All the three transcription factors displayed higher expression in monocytes compared to the other cell types and were upregulated in COVID-19 cases compared to healthy controls in both PBMC and whole blood datasets. For these transcription factors, the observed cell type-specific expression aligned with the regulons detected by REACTOR, supporting its ability to detect biologically relevant changes in transcription factor driven GRNs that remain robust across datasets. Overall, the results of this case study emphasize the importance of monocytes in COVID-19 responses, which is well in line with several earlier reports (Brauns *et al*. 2022, Junqueira *et al*. 2022, Maher *et al*. 2022).

### 3.2 Visualization and prioritization

The connectivity and shared target genes of the identified cell type-specific regulons can be visually inspected using e.g. Cytoscape (Fig. 1D). Furthermore, several additional custom criteria can be utilised in the inspection of the regulons, such as a cut-off based on the number of target genes per regulon or regulon importance values.

## 4 Discussion and conclusion

Several tools have been developed to study gene regulatory networks at the single-cell level, perhaps most notably the SCENIC framework (Aibar *et al*. 2017). We have developed an R package, REACTOR, which extends the existing GRN workflows by introducing a replicate-aware statistical framework that enables detection of differential regulon activity across experimental conditions, thereby addressing the limitations of currently available tools that lack this capability.

We demonstrated the use and performance of REACTOR with a publicly available COVID-19 dataset, where it successfully identified several differentially active regulons that have been previously linked to the disease in the literature. In case REACTOR outputs a large number of significant regulons across multiple cell types, the results are best explored through cell type-specific ranked lists rather than a single combined list. In the analysis design, users can define the clustering resolution of interest, for example, by searching for regulons within major cell types or within specific subpopulations. Furthermore, while REACTOR currently depends on SCENIC for estimating regulon activities, it can in the future be extended to take inputs from other GRN inference tools.

In conclusion, REACTOR provides a statistical framework for analyzing differential regulon activity in scRNA-seq datasets, filling a gap in the current single-cell GRN analysis tools. Overall, we anticipate that REACTOR will be useful for studies aiming at understanding cell type-specific regulatory mechanisms that underpin cellular functions and their disturbances in various healthy and disease states.

## Supplementary Material

btag203_Supplementary_Data

## Data Availability

REACTOR is freely available through GitHub at https://www.github.com/elolab/REACTOR. Processed count matrices for the COVID-19 dataset with de-identified metadata and embeddings are available for download from the COVID-19 Cell Atlas hosted by the Wellcome Sanger Institute (https://www.covid19cellatlas.org/#wilk20).
