# Supplementary material for: REACTOR: REgulon Activity analysis and Comparison Tool for single-cell transcriptOmics Research
Source: Bioinformatics. 2026 May 4;42(5):btag203. doi: 10.1093/bioinformatics/btag203 (PMC13218382; doi:10.1093/bioinformatics/btag203)
Supplement: btag203_Supplementary_Data [file btag203_supplementary_data.pdf]

Supplementary data for

REACTOR: REgulon Activity analysis and Comparison Tool for single-cell transcriptOmics Research

Markus Lindén[1]*, Sebastián I. Zúñiga Norman[1]*, Tommi Välikangas[1],
Sini Junttila[1,2], Tomi Suomi[1,#], Kalle T. Rytkönen[1,3,#], Laura L. Elo[1,3,#]
[1]Turku Bioscience Centre, University of Turku and Åbo Akademi University, Turku, Finland
[2]Faculty of Science, University of Turku, Turku, Finland
[3]Institute of Biomedicine, University of Turku, Turku, Finland
*Shared first authors
[#]Corresponding authors, email: TS: tomi.suomi@utu.fi, KTR: katury@utu.fi, LLE: laura.elo@utu.fi.

We used the COVID-19 resource (Välikangas et al. 2022, https://elolab.shinyapps.io/COVID19/) to investigate the expression of MAFG (**Supplementary Figure S1**), BCL6 (**Supplementary Figure S2**), and FOXN2 (**Supplementary Figure S3**) in nine independent datasets. All the three transcription factors displayed higher expression in monocytes (Lee at al. 2020) compared to the other cell types and were upregulated in COVID-19 cases compared to controls in both PBMC and whole blood datasets in the resource.

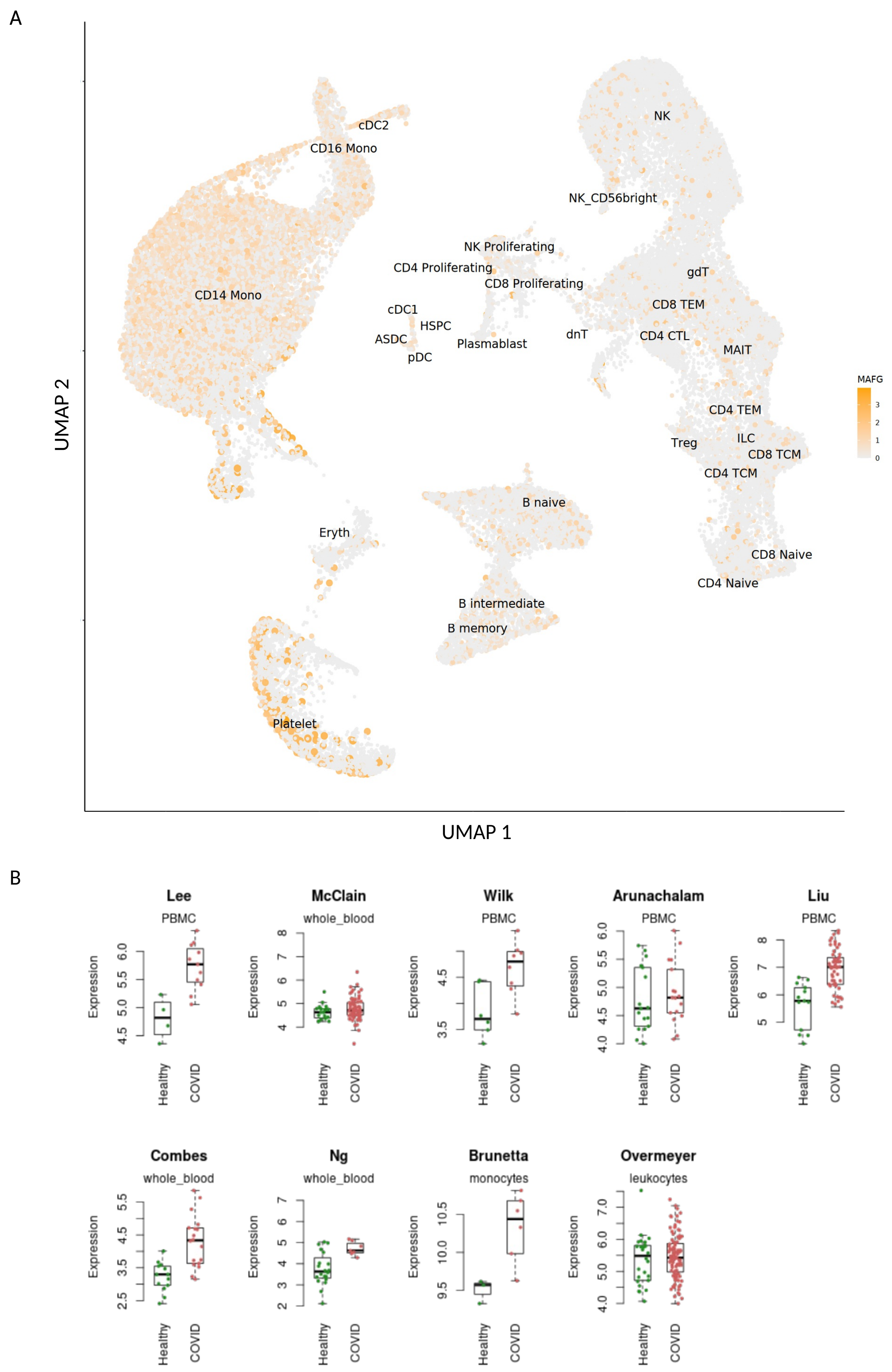

Supplementary Figure S1. **(A)** Single-cell view of MAFG from the COVID-19 resource (Välikangas et al. 2022) using the Lee dataset (Lee at al. 2020). **(B)** Boxplots summarizing

MAFG expression profiles in COVID-19 cases and healthy controls across all the available nine datasets.

A

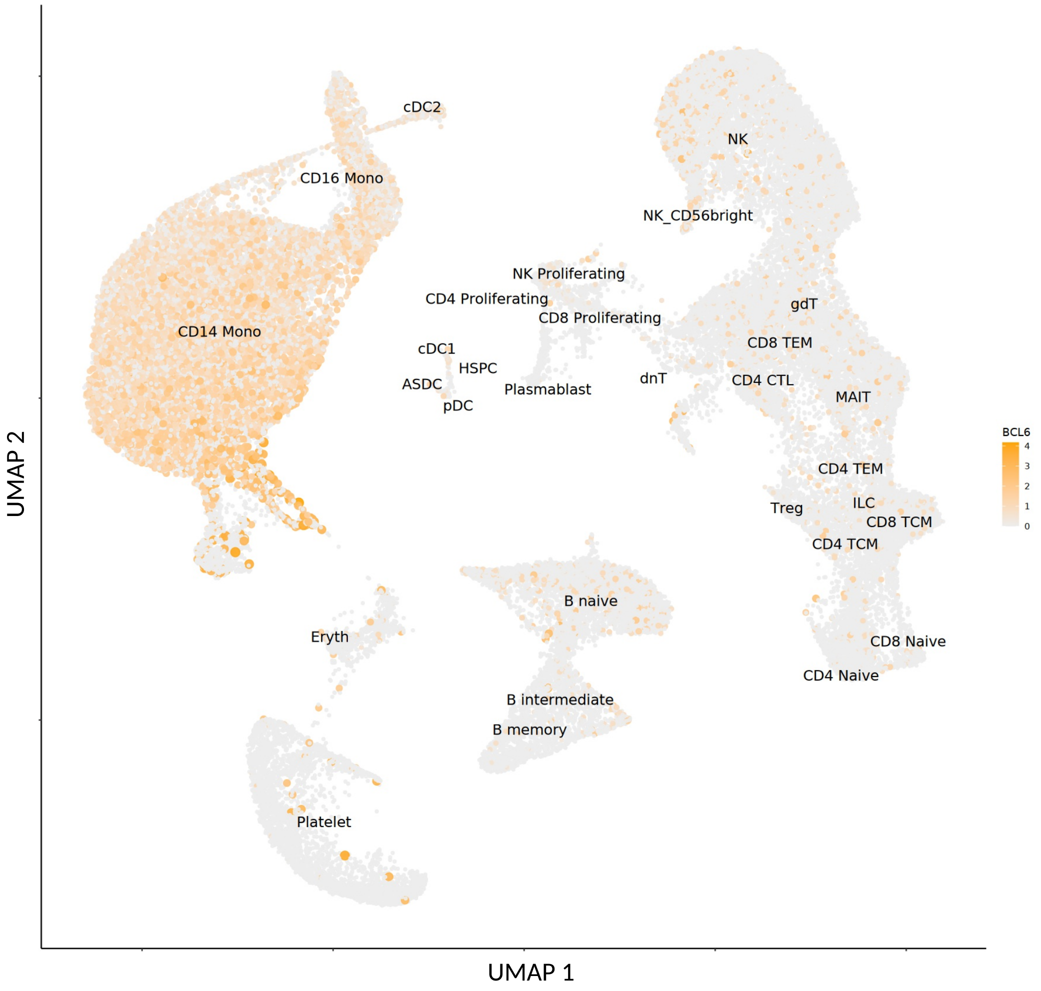

B

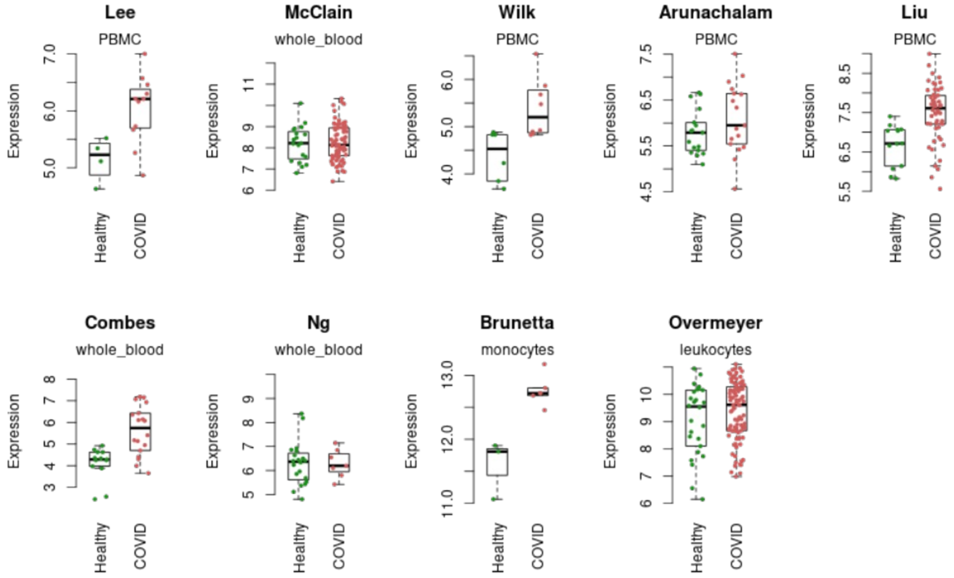

Supplementary Figure S2. **(A)** Single-cell view of BCL6 from the COVID-19 resource (Välikangas et al. 2022) using the Lee dataset (Lee at al. 2020). **(B)** Boxplots summarizing BCL6 expression profiles in COVID-19 cases and healthy controls across all the available nine datasets.

A

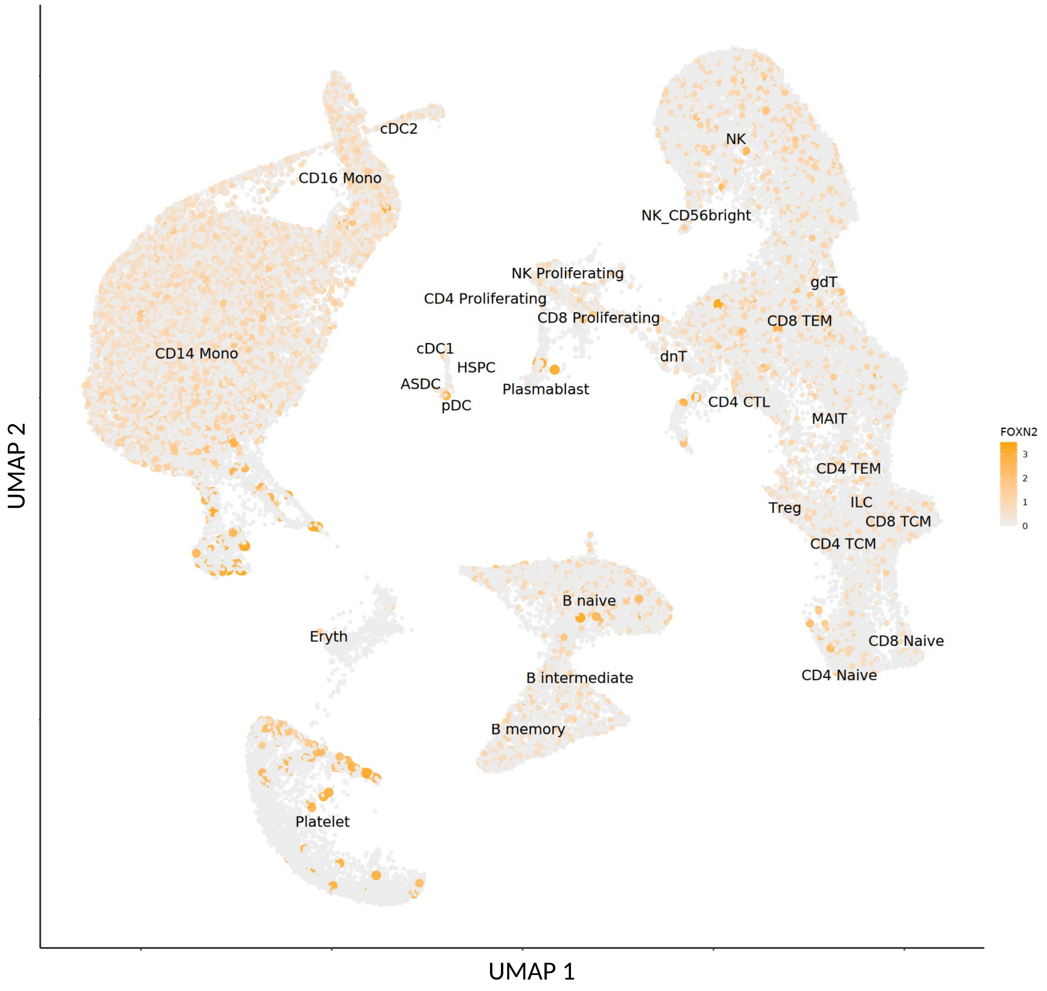

B

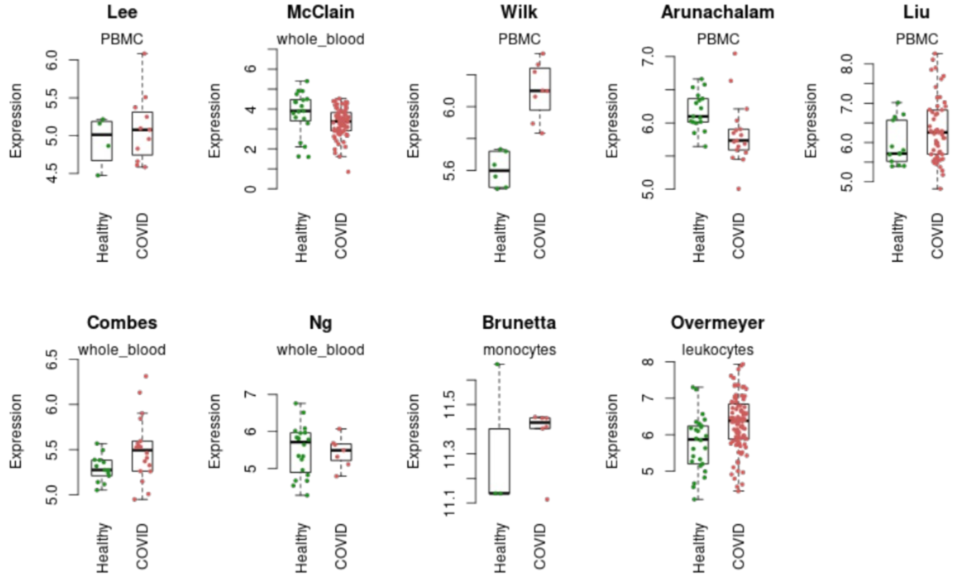

Supplementary Figure S3. **(A)** Single-cell view of FOXN2 from the COVID-19 resource (Välikangas et al. 2022) using the Lee dataset (Lee at al. 2020). **(B)** Boxplots summarizing

FOXN2 expression profiles in COVID-19 cases and healthy controls across all the available nine datasets.

References

Välikangas T, Junttila S, Rytkönen KT *et al.* COVID-19-specific transcriptomic signature detectable in blood across multiple cohorts. *Front Genet* 2022;**13**.

Lee, J. S. *et al.* Immunophenotyping of COVID-19 and influenza highlights the role of type I interferons in development of severe COVID-19. *Sci Immunol* 2020;**5**(49):eabd1554
